# Rewilding by Wolf Recolonisation, Consequences for Ungulate Populations and Game Hunting

**DOI:** 10.3390/biology11020317

**Published:** 2022-02-16

**Authors:** Mariano Rodríguez-Recio, Camilla Wikenros, Barbara Zimmermann, Håkan Sand

**Affiliations:** 1 Unit of Biodiversity and Conservation, Department of Biology and Geology, Physics and Inorganic Chemistry, Rey Juan Carlos University, ESCET, Tulipán s/n, 28933 Madrid, Spain; 2 Grimsö Wildlife Research Station, Department of Ecology, Swedish University of Agricultural Sciences, SE-73993 Riddarhyttan, Sweden; Camilla.Wikenros@slu.se (C.W.); Hakan.Sand@slu.se (H.S.); 3 Faculty of Applied Ecology, Agricultural Sciences and Biotechnology, Campus Evenstad, Inland Norway University of Applied Sciences, 2480 Koppang, Norway; barbara.zimmermann@inn.no

**Keywords:** trophic rewilding, human-predator conflicts, wolf recolonisation, game hunting, wolf predation, prey-species selection

## Abstract

**Simple Summary:**

Humans extirpated the wolf *Canis lupus* from many regions of Europe. Today, the wolf is returning to many of these areas, and with it, people’s opposition due to its predatory habits on, among others, ungulate game species. Based on existing data on wolf prey selection, kill rates and territory size, we extrapolated the results from central Sweden and Poland to southern Sweden, where wolf recolonization has not yet occurred and conservation conflicts with hunters are expected. Thus, we calculated the proportion of moose *Alces alces*, roe deer *Capreolus capreolus*, red deer *Cervus elaphus*, fallow deer *Dama dama* and wild boar *Sus scrofa* that would be killed by wolves in the municipalities of southern Sweden if wolf recolonization occurs. We found that the current system of five ungulate species in southern Sweden could potentially support a wolf density two to four times higher than in the current wolf distribution in central Sweden, which are mainly inhabited by roe deer and moose. With this type of research, we can anticipate and work to ameliorate the social unrest and expected conservation conflicts that may arise once wolves or other large carnivore species recolonize areas of Europe that are returning to the wild.

**Abstract:**

The ongoing recolonisations of human-transformed environments in Europe by large carnivores like the wolf *Canis lupus* means that conservation conflicts could re-surface, among other reasons, due to predation on ungulate game species. We estimated the effect of wolves on ungulate species using data on wolf prey selection, kill rates and territory size to build a hypothetical case of future expansion. We extrapolated results on predation from the current wolf distribution in central Sweden and eastern Poland to the eventual wolf recolonisation of southern Sweden. We then calculated the proportion of five ungulate game species killed annually by wolves, and the ratio between the predicted annual predation by wolves given future colonization and the number of ungulates currently harvested by hunters. Results showed that wolf recolonization in southern Sweden would have a minor impact on the estimated population densities of red deer *Cervus elaphus*, fallow deer *Dama dama* and wild boar *Sus scrofa*, but is likely to lead to a significant reduction in human captures of moose *Alces alces* and roe deer *Capreolus capreolus*. The current five-ungulate species system in southern Sweden suggests a potential for two to four times higher wolf density than the two-ungulate species system in the northern part of their current distribution. Management and conservation of recolonizing large carnivores require a better understanding of the observed impact on game populations under similar ecological conditions to ameliorate conservation conflicts and achieve a paradigm of coexistence. Integrating these predictions into management is paramount to the current rewilding trend occurring in many areas of Europe or North America.

## 1. Introduction

Rewilding promotes the recovery of natural processes, species and ecological functioning in human-transformed environments while reducing the impact of human activities [1]. However, rewilding can be controversial when it involves conflictual species, such as large carnivores [2]. Despite long persecution, some large carnivore species are currently recolonizing areas of their historic range where they were extirpated [3,4,5]. This recolonisation has been facilitated by conservation strategies, strict species-protection status [3], and the progressive rural exodus of people to cities due to socioeconomic reasons [6] that ultimately enable rewilding [7,8,9,10]. The recovery of iconic top-predators is a relevant milestone for initiatives such as Rewilding Europe (https://rewildingeurope.com, accessed 12 February 2022), which promotes the re-establishment of natural ecological processes and wilderness as an opportunity for sustainable business and employment [9,11]. However, the return of predators also implies the resurgence of old conflicts and new challenges inherent to the current characteristics of the recolonized environment [12,13].

The space and habitat requirements of large carnivores and their predatory behaviour as top predators make these species particularly vulnerable to human-induced mortality [3,4,12]. Humans often perceive large predators as a threat to themselves and their interests [4,14]. This perception and derived conservation conflicts stem from fear of attacks on humans [15,16], depredation on domestic animals (e.g., livestock and dogs) [7,17] and competition for shared resources such as game species [18]. The latter, particularly wild ungulates, are game species of economic and recreational importance to humans. Wild ungulates are also the staple prey of large carnivores such as the wolf (*Canis lupus*), an iconic predator strongly associated with societal and conservation conflicts [19,20,21]. The wolf is a generalist that occupies varied habitat types, preys upon a broad range of species, and has a large capacity for population growth [22,23]. As a top predator, the wolf can significantly impact prey dynamics with cascading effects on ecosystems, given free expansion and population growth [24]. Consequently, the wolf plays a relevant role in the rewilding process that is taking place in many areas where it was extirpated but is now returning. In Europe, the wolf is currently recolonizing historical regions of its distribution range [3,25], for example, in Scandinavia [26], Finland [18,27], Poland [28], Germany [13,29], Denmark [30] and Spain [7,31,32]. This return can result in competition for ungulate game species with hunters and trigger conservation conflicts [18,33,34]. Research capable of identifying the potential effects of wolf return on the ungulate community and the consequences for sustainable game management is needed to assist conservation and management decisions.

Research on wolf-ungulate dynamics has generally focused on predation impact on a primary prey species such as the caribou (*Rangifer tarandus*) [35], moose (*Alces alces*) [34,36], white-tailed deer (*Odocoileus virginianus*) [37] and red deer (*Cervus elaphus*) [38]. However, prey selection and its mechanisms within multi-prey systems have been less studied (but see [39,40,41,42]). In general, the abundance and size of different prey species and their vulnerability to predation are the main factors of prey selection by wolves [43]. This information can assist in informing and forecasting the impact of wolves on different prey species after recolonisation. Here, we used data on wolf prey selection, kill rates and territory size identified in the current wolf distribution range in central Scandinavia [36,44] and eastern Poland [38,45,46] to analyze the potential impact of this predator on the populations of five-game ungulate species and the emerging consequences for game harvest in southern Sweden. Recent computer simulations based on observed recolonisation patterns predicted a very slow expansion of wolves into southern Sweden before 2030 [47]. During the 2020/2021 winter, two wolf pairs were established in this region [48], indicating incipient colonization of this area, which will most likely result in increased competition with game hunting. Our procedure and results represent a hypothetical scenario of the future expansion of wolves into this region [47] as a case study of other similar processes in Europe [49]. Based on ungulate and wolf densities and the size of wolf territories, we extrapolated available results on wolf predation in a community of two ungulate species to areas that included up to five ungulate species. We then conducted a predictive exercise to identify the potential impact on these five game ungulate species after the establishment of wolf territories based on the proportion of each ungulate species annually killed by wolves, wolf density, and the ratio between the predicted annual wolf predation and the number of ungulates harvested by hunters [47,50]. The community of ungulates in southern Sweden shows similarities with other areas of ongoing wolf recolonization in Europe, such as Poland, Germany or France. Therefore, our research can provide valuable information and insights on the expected impact of the wolf return on game species and the magnitude of resource competition with hunters. Understanding and forecasting the impact of returned predators like the wolf on game species will better prepare the management and conservation for rewilding initiatives.

## 2. Methods

### 2.1. Study Area

**Figure 1:**
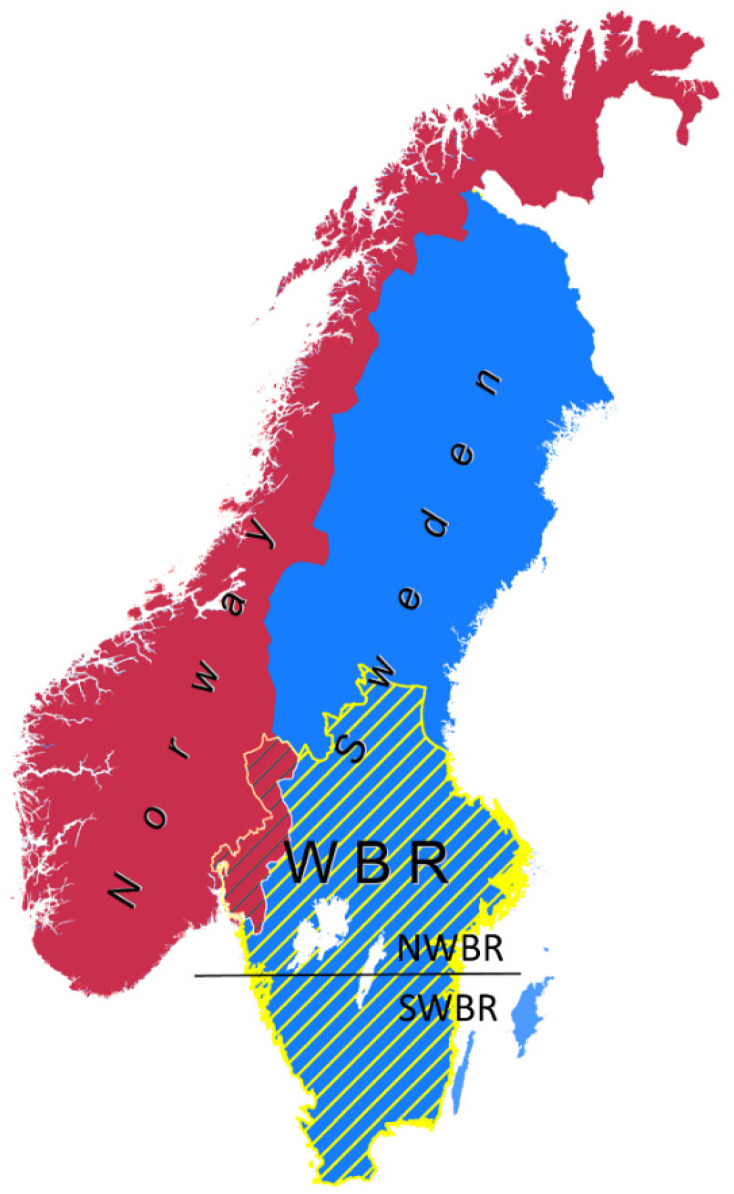
The study area in the Scandinavian Peninsula is highlighted with yellow lines depicting the wolf breeding range (WBR) in Sweden (blue background). Blue lines indicate the WBR in Norway (red background) not considered in this research. The horizontal black line indicates the division between the North and South of the WBR (NWBR and SWBR, respectively).

We focused on Central and Southern Sweden (Figure 1), hereafter referred to as the wolf breeding range (WBR, [26]). This area of the Scandinavian Peninsula in Sweden (and Norway) is where the wolf is allowed to establish territories and excludes the northern half of the country where reindeer husbandry occurs and management policies hinder wolf recolonization [26,47,51]. Therefore, the northern part of the WBR (NWBR) has been the main distribution range of the wolf population in Sweden. On the other hand, the southern part of the WBR (SWBR) includes a relatively higher density of the human population, agriculture (including domestic livestock), and traffic infrastructure than the NWBR [26].

### 2.2. The Wolf in Sweden

The wolf population in Sweden and Norway consisted of approximately ~480 individuals in the winter of 2020/2021 [48], mostly distributed in Sweden (~80%) and the bordering areas in east-central Norway (~20%). In Sweden, the wolf is classified as vulnerable [52] and protected under the Habitat Directive 92/43/ECC of the EU. Considered functionally extinct in the late 1960s, the initial immigration of wolves from Finland and Russia facilitated the recolonization of the peninsula in the early 1980s [50]. However, recolonization and population growth have been limited and constrained by various factors, including management (license and protective harvest), illegal killing (poaching) and inbreeding depression [33,50,53]. Under the management regime in Sweden up to 2019, the wolf population was allowed to expand into the remnant areas of NWBR or the whole SWBR (Figure 1). Although previous studies identified SWBR as a suitable habitat for wolf recolonization [26,51,54], this process has been slow and up to 2019, limited to only a few short-lived territories with no reproduction [47].

### 2.3. Ungulate Populations in WBR

The moose population has a fairly even distribution and density throughout Sweden, with a total population estimated at 265,000 individuals [55]. The roe deer (*Capreolus capreolus*) occurs at low or zero densities in the northern part of Sweden but shows a sharp increase in density in the WBR towards the south with a total estimated population of 650,000 [56]. Wild boar (*Sus scrofa*), red deer, and fallow deer (*Dama dama*) all inhabit the southern and eastern parts of the WBR, with populations steadily increasing in both range and density. The wild boar is currently recolonizing the south and eastern parts of the country after incidental reintroduction in the 1970s [57]. The total population in Sweden is estimated to be 150,000 individuals (last estimations from 2010) [57,58]. After near extinction in the 1960s, the red deer also recovered notably from a small remnant population in the southernmost parts of Sweden and was later reinforced by the introduction of non-Swedish individuals [59]. In 2016 the population was estimated to number 26,000 individuals [60]. The fallow deer was introduced into Sweden in the 16th century but has shown slow population growth, which in 2016 was estimated at 126,000 individuals [60,61].

### 2.4. Ungulate Density and Distribution

We prepared data on ungulate population density and distribution under two main assumptions. As harvest size is closely related to the population density of moose and roe deer in Scandinavia [62,63], we used the 2016 hunting bag data (individuals harvested/km^2^) to estimate the densities of the five ungulate species in each municipality of the WBR (*n* = 102, mean area = 649 km^2^, range = 87–1606 km^2^). Since our objective was to estimate prey density across municipalities for relative comparisons, our initial assumption (see Table S1 for a full list of assumptions adopted) considered a relationship between harvest size and the total population size of each ungulate species. However, because the potential for reproduction and population growth differs between ungulate species, we used a species-specific reproductive rate to calculate the actual population density from harvest data for each municipality. For municipalities where wolves were absent in 2015/2016, we assumed that harvest alone approximately equated the yearly reproductive rate of the ungulate populations. This assumption means that other types of mortality than harvest were accounted for and included in the reproductive rates used below. Thus, we acknowledge these rates are approximations, rather than estimates, at specific sites throughout the prey distribution range. The reproductive rates used for calculating the total population size of each ungulate species under the assumption above were 0.27 (i.e., 27% annual population growth) for moose [64], 0.16 for roe deer [65], 0.31 for red deer [66], 0.31 for fallow deer (assumed equal to red deer), and 0.40 for wild boar [67]. Thus, we estimated for each municipality the approximate density of each ungulate species and their proportions in the ungulate community (Supplementary Materials).

We then applied a second assumption that considered the sum of the registered annual harvest and the estimated wolf predation (in the municipalities where the wolf was present during the monitoring season 2015/2016) approximately equated the yearly reproductive rate in the prey population. This assumption meant harvest together with wolf predation and other sources of mortality (vehicle collisions and natural causes represent 7% of the total moose mortality within wolf territories [68]) roughly balanced prey populations at some level. This assumption was based on harvest data for moose and roe deer, which showed a temporally limited but spatially variable trend in total annual harvest size over the years before 2016 among management units (https://algdata-apps.lansstyrelsen.se/algdata-apps-stat, accessed 12 February 2022). The other three prey species have shown an increasing trend in the harvest, which suggests an annual production in the population larger than the total harvest [60]. However, we also applied our second assumption to these species acknowledging a most likely slight underestimate of the actual population size and population growth. We confirmed wolf territory presence in each municipality from the information given for the monitoring season 2015/2016 [69].

### 2.5. Prey Selection

We applied wolf selection ratios for the different ungulate prey species reported in previous studies conducted in Scandinavia [70] and Poland [40,46]. From these, we identified the selection ratios among the five ungulate species in SWBR. The selection ratio of preferences ranked from 1.5 for red deer, followed by moose and roe deer with 1.0 (assuming a selection proportional to the occurrence of each species; [70]), and 0.5 for the wild boar, i.e., selected less than the expected occurrence. Because no information on the selection ratio for fallow deer was available, we assumed the same value applied to the red deer due to their similar social organization and body size.

### 2.6. Wolf Kill Rates

We first used the wolf kill rate of moose in Scandinavia [36,44] to calculate kill rates of the remaining four prey species (See Supplementary Information for a detailed description of the calculations). These studies identified a wolf pack (≥2 individuals) killed on average 120 moose per year, which provided an edible biomass per wolf and day of 8.0 kg [71]. The average pack size (including ≥2 wolves) in Scandinavia is 4.26 [63], which results in an average total annual available biomass of 12,440 kg for a wolf pack that relies only on moose. We applied an average species-specific prey size for the other prey species and calculated kill rates related to their equivalence of moose. The average edible biomass (set to 65% of total body weight for all age and sex classes) used per wolf-killed individual of moose, roe deer, red deer, fallow deer and wild boar, was 114, 15, 65, 43 and 17 kg, respectively (H. Sand unpublished data, [36,44]). However, wolves in Scandinavia generally consume approximately only 70% (equal to 5,6 kg per individual/day) before they abandon killed moose carcasses, and the remaining biomass is consumed mainly by other species [68]. For the case of the other four ungulate species, we assumed consumption of 100% of the available biomass.

Jędrzejewski et al. [38] estimated an annual kill rate of 114 red deer, 54 wild boar and 8 roe deer per wolf pack in Poland, resulting in daily edible biomass of 5.3 kg per wolf and a total annual estimate of 8465 kg for an average pack of 4.4 wolves. Similarly, we based our calculations of total annual kill for the four smaller ungulate species than moose on a total annual estimate of edible biomass for an average wolf pack of 4.26 individuals to 8197 kg (equal to 5.3 kg per individual/day). Assuming that a wolf pack entirely relied on only one of the five prey species yielded an annual total kill per species of 120 moose, 518 roe deer, 143 red deer, 215 fallow deer, or 451 wild boars. We then adjusted our calculations so that any combination of the four smaller prey species equated the estimated annual total edible biomass available per pack (8197 kg). In contrast, for the contribution of moose to the total consumption, we used the annual estimate of 12,440 kg [36,44].

We assumed a capped type 1 functional response where the total annual kill of a prey species is linearly related to its relative abundance in the ungulate community and the level of saturation (cap) determined by the maximum annual total kill of each prey species. Thus, for each municipality, the number and composition of prey species killed by a wolf pack per year was the result of (1) the relative proportion of the particular prey species in the ungulate community, (2) the prey species-specific selection ratio, and (3) the estimated prey species-specific kill rate. This result allowed us to calculate the predicted total number of individuals of each ungulate species killed by wolves in all the municipalities per square kilometre. Then, we added to the initial estimate of the number of ungulates of each species (i.e., calculated from harvest) the number of individuals that wolves would take in each of these municipalities if they were present. This way, we converted the current scenario into the hypothetical scenario (i.e., that annual harvest approximately equated ungulate reproduction) also occurred with wolf presence in all the municipalities of the study area. This hypothetical scenario allowed forecasting the required reduction in harvest for each prey species and municipality needed to balance the estimated mortality due to wolf predation while making the simplifying assumption that wolf predation was entirely additive for harvest.

### 2.7. Estimating the Potential for Wolf Density

We estimated the potential wolf density per municipality, assuming the same pack size applied in previous calculations. Prey availability is considered an important driver of predator density [24] and wolf density is dependent on available ungulate biomass [22,37]. In addition, previous research in Scandinavia showed wolf territory size was negatively related to the density of roe deer and positively to latitude [63]. Thus, we used the Mattisson et al. [63] model to estimate the potential wolf territory size in each municipality, applying the combined density of the three deer species other than the moose and assuming these ungulates would all contribute to determining the wolf territory size [45]. We also set a lower limit for territory sizes of 259 km^2^, corresponding with the smallest territory size identified so far in Scandinavia [47,63]. We then calculated the maximum number of wolf territories that could fit into a municipality by dividing the municipality area by the estimated wolf territory area.

### 2.8. Combining Prey Density, Predator Density and Kill Rates

Finally, we combined kill rates per ungulate prey species and the number of wolf territories per municipality to estimate the predation rate (*Pr1*) for each ungulate species. This rate was the estimated proportion of each ungulate population killed by wolves per municipality. We also estimated the potential competition for ungulates between wolves and hunters by calculating for each ungulate species the ratio of estimated wolf predation to actual harvest (*Pr2*). This ratio was equal to the number of harvested individuals of each ungulate species divided by the estimated number killed by the wolf. A *Pr2* > 1 indicated wolves are likely to kill more prey species than the prey population could sustain. Therefore, we would expect a reduction in prey density even after a total cessation of harvest.

## 3. Results

**Figure 2:**
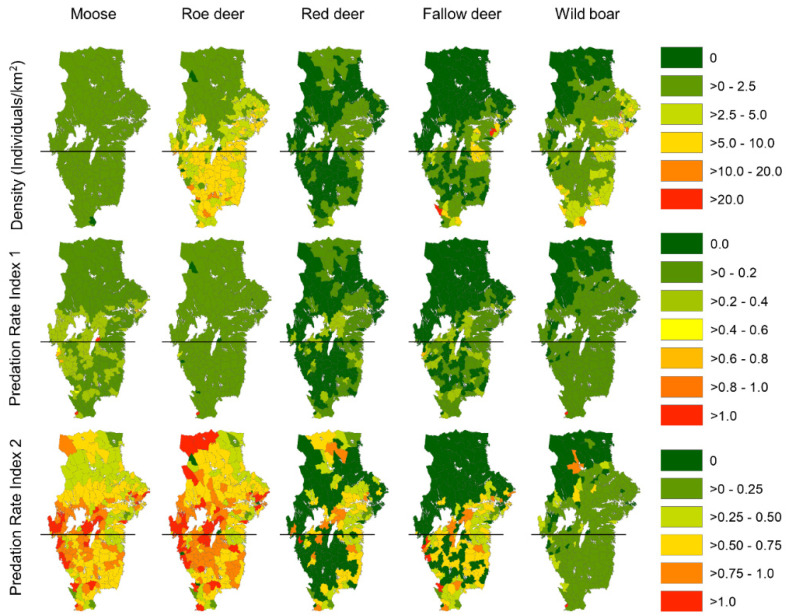
Maps on the density of each ungulate species per municipality quantified from hunting bags in 2016/2017 (top row). Maps in the middle row depict the predation rate index 1 *(Pr1*) per municipality. This index was calculated as the estimated number of ungulate individuals per species killed by wolves divided by the estimated number of that ungulate species in the municipality. The maps below show the predation rate index 2 (*Pr2*). This index was the ratio between the number of individuals per ungulate species killed by wolves divided by the number of individuals of each ungulate species harvested by hunters. Horizontal black lines separate the north (NWBR) and south (SWBR) of the wolf breeding range (WBR).

Moose density was homogenously distributed all over WBR (mean ± 95%CI = 0.78 ± 0.04; range 0.1–1.5 individuals/km^2^ in the municipalities), and roe deer showed an increasing trend in population density from north to south with higher densities in SWBR (mean ± 95%CI = 5.67 ± 0.51; range = 0–14 individuals/km^2^) than in NWBR (mean ± 95%CI = 2.95 ± 0.39; range = 0–10 individuals/km^2^, Figure 2). Red deer and fallow deer were mainly distributed in the southeastern parts of WBR, with a variation between municipalities ranging from 0 to 5 and from 0 to 10 individuals/km^2^, respectively. A similar geographical distribution also occurred for the wild boar (0–5 individuals/km^2^), with the highest densities located in the southeastern-most areas of WBR. Overall, the total population biomass of the ungulate species was 1.3 times higher in SWBR (mean ± 95%CI = 489 ± 13 kg/km^2^; 102 municipalities) than in NWBR (374 ± 10 kg/km^2^; 149 municipalities), although with relatively large variation between municipalities within each region (NWBR range = 38–1419 kg/km^2^, SWBR range = 26–1669 kg/km^2^). However, the biomass of deer species (roe deer, red deer, fallow deer) in SWBR was 1.17 times higher than in NWBR.

**Figure 3:**
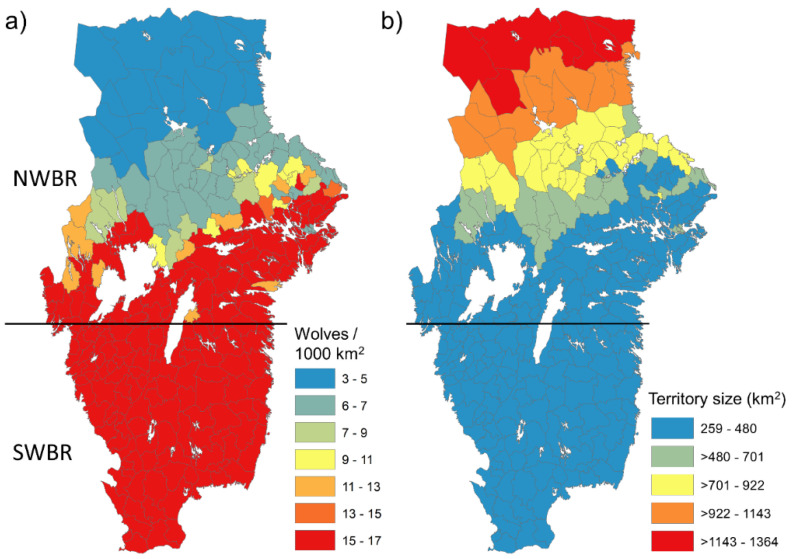
Predictions on wolf numbers and territory sizes obtained in the Swedish wolf breeding range (WBR), divided as north (NWBR) and south (SWBR). (**a**) Estimated number of wolves per 1000 km^2^ obtained from the number of wolf territories that the abundance of deer prey (roe deer, red deer, fallow deer) can sustain per municipality and considering that a wolf territory contains on average 4.26 wolves in Scandinavia [63]. (**b**) Estimated wolf territory size (km^2^) per municipality as a function of deer density and latitude as modelled in Mattisson et al. [63].

As indicated from the geographical variation in prey (deer) density, calculations on the potential number of resident wolves (pairs and packs) predicted from deer density, latitude and wolf territory size showed a southward gradient of an increasing number of wolves per 1000 km^2^ (Figure 3). The relatively higher densities of deer in the southern part of NWBR and throughout the SWBR resulted in wolf densities that may reach 15–17 resident wolves/1000 km^2^ compared to 3–5/1000 km^2^ in the northern part of the NWBR. Conversely, wolf territories in the northern part of the NWBR averaged 1000 km^2,^, whereas predicted territory sizes in the SWBR ranged between 260 and 480 km^2^ (Figure 3).

The predicted predation rate (*Pr1*), estimated as the average (between municipalities) proportion of each ungulate species annually killed by wolves in the NWBR, was highest for moose, followed by roe deer, wild boar, red deer and fallow deer. *Pr1* was similar in the SWBR except for a reversed order for red deer and fallow deer (Table 1).

The predation-to-harvest ratio (*Pr2*) indicated the wolf recolonization of NWBR and SWBR will result in a considerably lower (<0.30) annual wolf predation than the numbers killed by hunters for red deer, fallow deer, and wild boar (Table 1). Conversely, for moose and roe deer, the estimated predation-to-harvest ratio indicated a markedly higher potential for competition with human harvest (Table 1). For all prey species except for red deer, *Pr2* was slightly higher in the SWBR than in the NWBR. Both estimates of predation impact showed a large spatial variation between municipalities (Figure 3).

## 4. Discussion

We used observed patterns of wolf predation and recolonization to quantify and predict the potential impact on ungulate species and game hunting caused by the expected wolf recolonization of areas in Sweden. Our results show that the biological potential for the highest wolf densities lies outside the current wolf range in Sweden. Moreover, the current prey availability in the southern parts of the NWBR and most of the SWBR will allow for higher or much higher densities of wolves than those found in the current distribution range. Our results also reveal that an expansion of the wolf population into the SWBR would have a minor impact on the estimated population densities and size of the sustainable harvest for three of the five ungulate species (red deer, fallow deer, wild boar). However, for moose and roe deer, the establishment of wolf territories is likely to result in a general reduction in the potential for a sustainable and local game harvest for hunters.

Our results are supported by a recent study on the impact of wolves on moose harvest in the NWBR, which showed wolf recolonization might decrease harvest by >50% compared to areas without wolves [34]. Our predictions indicate the strongest effect of wolf predation on moose may occur in the southwestern parts of the WBR, where the prey community consists of moose, roe deer and wild boar but not of red deer and fallow deer. Although relatively high densities of roe deer and wild boar can dilute wolf predation for the remaining three available ungulate species, it may also potentially facilitate higher wolf densities by reducing wolf territory size [22,45,63]. This scenario may be viewed as an apparent competition where high densities of alternative prey (roe deer and wild boar) may result in a higher predation rate on moose, a less abundant prey but equally important for wolves (by kg biomass) [72]. Conversely, the areas with higher densities of red deer and fallow deer in the southeast are likely to result in a lower predation impact on moose and roe deer. This is because the former are more strongly selected by wolves [40,46] and contribute to a more diverse prey community in this area.

The SWBR was previously identified as highly suitable for wolves based on biotic and anthropogenic factors [26,51]. However, this region also has a higher mortality risk due to traffic, poaching, and legal harvest [26]. With the observed pattern of wolf expansion, which has been largely shaped by active management through a selective harvest regime during the last decades in Sweden, it is unlikely that all SWBR be colonized during the next 10–15 year period [47]. However, if wolf expansion is facilitated in SWBR, a clumped pattern of establishment with multiple hot spots of one to several adjacent wolf territories would be the most likely pattern to occur shortly, as a continuation of the recent establishment of two territorial pairs in SWBR identified during the 2020/2021 monitoring season [48]. This trend highlights the timely importance of this research as a guide for general predation patterns after wolf recolonization. In addition to the effect on game species, depredation on domestic sheep is anticipated as sheep are more abundant in this part of Sweden [26,51]. Consequently, conflicts of interest concerning competition for game and depredation on domestic animals can ultimately lead to increased legal and illegal wolf culling and hamper recolonization [33].

We argue that our model results should be mainly perceived as an exercise to predict general spatial patterns rather than absolute estimates for single municipalities. We acknowledge there may be individual/wolf pack differences in prey species preference, kill rates and space use beyond our considerations. The same applies to our estimates of prey population growth and size. We also used several assumptions in our model, including that our results are based on a static system; that is, what would be the initial and most likely outcome once wolves become established in this recolonizing area. However, biological systems are generally dynamic, and prey and predator populations may fluctuate over time, making long-term predictions more uncertain. In particular, for this system with a high anthropogenic impact on wolf prey populations, the functional response of hunters to adjust the size of harvest proactively following the local establishment of wolves is a crucial component. In Sweden, hunters have responded to wolf return by reducing harvest and/or changing the composition of harvested animals to compensate for wolf predation in NWBR [34]. To achieve a paradigm of coexistence with large carnivores [2], hunters need to accept sharing game species with the wolf [64]. A shift towards this paradigm can be encouraged by implementing effective rewilding initiatives to reverse defaunation, increase biodiversity, and restore natural functions, including trophic processes [8], wherein predators occupy a core and essential role. In this vein, our research exemplifies how using available empirical data obtained from similar or close ecological conditions can contribute to forecasting the potential impact of a large carnivore. This knowledge can contribute to more constructive discussions rather than heated debates about conservation conflicts [5,18,73]. This approach is particularly critical in the current rewilding trend occurring in many depopulated areas once severely impacted by longstanding human activities in Europe or North America and where local fauna, including predators, are returning [2,74,75,76]. Consideration of observed patterns of predator recolonization, as in the case of the wolf, and the predictions derived from them will help decision-making of local and transboundary policies [7,47].

## 5. Conclusions

Applying previous knowledge on the predatory behaviour of large provides essential insights into the future development of human-carnivore conflicts. This knowledge is particularly important for anticipating the impact of ongoing recolonizations of large carnivores on game harvest, as large carnivores and humans tend to use the same type of ungulate species. In doing so, the management of large carnivores and game harvest can be proactive to lessen conservation conflicts. Managers can better anticipate and inform the public of large carnivore recolonization’s most likely outcome and consequences. For the Scandinavian case, areas still to recolonize in the SWBR can potentially harbour higher densities of wolves than outside the current wolf distribution range. The wolf recolonization of the SWBR would likely have a minor impact on the sustainable harvest of red deer, fallow deer, and wild boar but lead to a general local reduction of moose and roe deer.

## Figures and Tables

**Table 1:** Mean ± 95% Confidence intervals of the predation ratio (*Pr1*) and predation-to-harvest ratio (*Pr2*) for each ungulate species in the north (NWBR) and south (SWBR) of the total of the wolf breeding range in Sweden.

	*Pr1*			*Pr2*		
	NWBR	SWBR	Total	NWBR	SWBR	Total
Moose	0.17 ± 0.04	0.20 ± 0.05	0.18 ± 0.03	0.65 ± 0.14	0.74 ± 0.18	0.69 ± 0.11
Roe deer	0.13 ± 0.01	0.19 ± 0.01	0.15 ± 0.01	0.92 ± 0.04	1.17 ± 0.04	1.02 ± 0.03
Red deer	0.05 ± 0.01	0.04 ± 0.05	0.05 ± 0.02	0.16 ± 0.12	0.13 ± 0.29	0.15 ± 0.14
Fallow deer	0.04 ± 0.01	0.09 ± 0.02	0.06 ± 0.01	0.13 ± 0.03	0.28 ± 0.06	0.19 ± 0.03
Wild boar	0.10 ± 0.01	0.17 ± 0.05	0.13 ± 0.02	0.31 ± 0.05	0.43 ± 0.11	0.36 ± 0.05

## Data Availability

Not applicable.

## Supplementary Materials

The following supporting information can be downloaded at: https://www.mdpi.com/article/10.3390/biology11020317/s1, Supplementary Material. Text: Formulas applied to calculate ungulate density and distribution, as well as prey selection and wolf kill rates. Table S1. Description of the assumptions made in the research, the area of origin of the data that justified the assumptions (if any) and the references to the articles that supported them (if any).
