# Supplementary material for: Rewilding by Wolf Recolonisation, Consequences for Ungulate Populations and Game Hunting
Source: Biology (Basel). 2022 Feb 16;11(2):317. doi: 10.3390/biology11020317 (PMC8869524; doi:10.3390/biology11020317)
Supplement: Supplementary file 1 [file biology-11-00317-s001.zip › biology-1523201-supplementary.pdf]

SUPPLEMENTARY MATERIAL

Rewilding by wolf recolonisation, consequences for game ungulate populations and game hunting

Mariano Rodríguez-Recio, Camilla Wikenros, Barbara Zimmermann, Håkan Sand

- Ungulate density and distribution

Ungulate species u_n:

u_1 = Moose
u_2 = Red deer
u_3 = Fallow deer
u_4 = Roe deer
u_5 = Wild boar

The estimated total number of individuals per municipality of each ungulate species above (Nu_n) resulted from applying to the number of harvested individuals of each species (Hu_n) a conversion factor on the reproductive rates per species cf_n. This factor was equal to 0.27 for moose (Jonzén et al., 2013), 0.31 for red and fallow deer (Gaillard et al., 2000), 0.16 for roe deer (Melis et al., 2013), and 0.4 for wild boar (Massei et al., 2015):

$$Nu_n = \frac{Hu_n}{cf}$$

The density of each ungulate species per municipality (du_n) was calculated by dividing the number of individuals of each species in each municipality (Nu_n) by the area of each municipality. Thus, the total ungulate density per municipality (T_{du}) was:

$$T_{du} = \sum_{n=1}^{5} du_n$$

The proportion of the *n* ungulate species in the community (Pu_n) resulted from:

$$Pu_n = \frac{du_n}{T_{du}}$$

- Prey selection and wolf kill rates

The average edible ungulate biomass available to wolves per individual of each ungulate species u_n (bu_n) was estimated as 114 kg for moose, 65.1 for red deer, 43.4 for fallow deer, 15 kg for roe deer, 17.25 for wild boar (H. Sand unpublished data, Sand et al. 2008, Zimmermann et al. 2015). Thus, the total biomass of each ungulate species u_n available to wolves per municipality (Tb_{u_n}) was:

$$Tb_{u_n} = Nu_n \times bu_n$$

, and the total biomass of ungulates available to wolves per municipality (Tb_u) was:

$$Tb_u = \sum_{n=1}^{5} Tb_{u_n}$$

Thus, the proportion of total biomass per ungulate species u_n available to wolves (Pb_{u_n}) was:

$$Pb_{u_n} = \frac{Tb_{u_n}}{Tb_u}$$

However, an average wolf pack size of 4.26 individuals in Scandinavia would require under the exclusive presence per municipality of one of the ungulate species u_n a total biomass ($Pack\_b_{u_n}$) of 8197 Kg per year of either roe deer, red deer, fallow deer, or wild boar, or 12442 kg of moose (due to the fact that moose was consumed on average only by 70% and the assumption that the other smaller ungulate prey species were consumed to 100%). Moreover, under a theoretical equal availability of the ungulate species present in a given municipality and an equal preference of selection by wolves, these would consume the proportional part of the 12442 kg of moose, if this species is present, and of the 819**7** kg of the rest of any of the other ungulates species present in the municipality. Thus, considering the availability in the municipality of the species u_n as 1 (presence) or 0 (absence), the theoretical proportional biomass of each species to a wolf pack considering equal availability (α) was:

$$\alpha_{u_n} = Pack\_b_{u_n} \times \sum_{n=1}^{5} \frac{1}{Availability(u_n)}$$

Because wolves select some prey species over others, we applied the following identified selection ratio reported in (Sand et al., 2016) and (Jędrzejewski et al., 2012) for each ungulate species u_n (s_{u_n}):

- Moose: s_{u_1} = 1
- Red deer: s_{u_2} = 1.5
- Fallow deer: s_{u_3} = 1.5
- Roe deer: s_{u_4} = 1
- Wild boar: s_{u_5} = 0.5

Because variations in the selection ratio depending on the species densities means different proportions of prey species killed, it is required to adjust the number of individuals killed depending on the composition of prey species. Thus, considering the selection ratio and different availability of each ungulate species given by Pb_{u_n}, the final available biomass per ungulate species u_n and municipality for a wolf pack (β_{u_n}) was:

$$\beta_{u_n} = Pack\_b_{u_n} \times Pb_{u_n} \times s_{u_n}$$

, that resulted in Ku_n number of ungulates killed per wolf pack, year, and municipality:

$$Ku_n = \beta_{u_n} \times bu_n$$

- Assumptions

Table S1.- Description of the assumptions made in the research, the area of origin of the data that justified the assumptions (if any) and the references to the articles that supported them (if any).

	**Assumptions**	**Data sources**	**References**
Ungulate population size	A1.- A relationship between harvest size and the total population size of each ungulate species.	A1.- Sweden	A1.- Mattisson et al. (2013); Ueno et al. (2014)
Ungulate density	A2.- Harvest alone approximately equated the yearly reproductive rate of the ungulate populations.<br>A3.- The reproductive rates for fallow deer were equal to red deer.<br>A4.- The sum of the registered annual harvest and the estimated wolf predation approximately equated the yearly reproductive rate in the prey population (i.e. harvest + wolf predation and other sources of mortality roughly balanced prey populations at some level).	A2.- Sweden and Europe.<br>A4.- Based on data on moose and roe deer in Sweden.	A2.- The reproductive rates were taken from Jonzén et al. (2013) for moose, Melis et al. (2013) for roe deer, Gaillard et al., (2000) for red deer, and Massei et al. (2015) for wild boar.<br>A4.- Wikenros et al., (2019), https://algdata-apps.lansstyrelsen.se/algdata-apps-stat
Prey selection	A5.- Prey selection ratios in Scandinavia were equal to those in Poland and assumed a selection proportional to the occurrence of the species.<br>A6.- Selection for roe deer was equal to that of red deer.	A5.- Scandinavia and Poland	A5.- Sand et al. (2016) and Poland (Jędrzejewski et al. (2000, 2012)
Wolf kill rates	A7.- Consumption of 100% of the available biomass for all the ungulate species but for the moose.<br>A8.- The daily edible biomass for the ungulate species other than moose in Sweden was the same than in Poland, i.e. 5.3 kg per wolf.<br>A9.- Capped type 1 functional response: the total annual kill of a prey species is linearly related to its relative abundance in the ungulate community, and the level of saturation (cap) determined by the maximum annual total kill of each prey species.	A8.- Poland	A8.- Jędrzejewski et al. (2002)
Estimating wolf density	A10.- The three deer species other than the moose contributed to determining the wolf territory size.	A10.- Sweden	A10.- Mattisson et al. (2013), Jędrzejewski et al. (2007)

REFERENCES

Gaillard, J.M., Festa-Bianchet, M., Yoccoz, N.G., Loison, A., Toïgo, C., 2000. Temporal Variation in Fitness Components and Population Dynamics of Large Herbivores. Annu. Rev. Ecol. Syst. 31, 367–393.

Jędrzejewski, W., Jedrzejewska, B., Okarma, H., Schmidt, K., Zub, K., Musiani, M., 2000. Prey Selection and Predation by Wolves in Bialowieza Primeval Forest, Poland. J. Mammal. 81, 197–212.

Jędrzejewski, W., Niedziałkowska, M., Hayward, M.W., Goszczyński, J., Jędrzejewska, B., Borowik, T., Bartoń, K.A., Nowak, S., Harmuszkiewicz, J., Juszczyk, A., Kałamarz, T., Kloch, A., Koniuch, J., Kotiuk, K., Mysłajek, R.W., Nędzyńska, M., Olczyk, A., Teleon, M., Wojtulewicz, M., 2012. Prey choice and diet of wolves related to ungulate communities and wolf subpopulations in Poland. J. Mammal. 93, 1480–1492.

Jędrzejewski, W., Schmidt, K., Theuerkauf, J., Jędrzejewska, B., Kowalczyk, R., 2007. Territory size of wolves *Canis lupus*: Linking local (Białowieża Primeval Forest, Poland) and Holarctic-scale patterns. Ecography 30, 66–76.

Jędrzejewski, W., Schmidt, K., Theuerkauf, J., Jędrzejewska, B., Selva, N., Zub, K., Szymura, L., 2002. Kill rates and predation by wolves on ungulate populations in a Białowieża primeval forest (Poland). Ecology 83, 1341–1356.

Jonzén, N., Sand, H., Wabakken, P., Swenson, J.E., Kindberg, J., Liberg, O., Chapron, G., 2013. Sharing the bounty—Adjusting harvest to predator return in the Scandinavian human–wolf–bear–moose system. Ecol. Modell. 265, 140–148.

Massei, G., Kindberg, J., Licoppe, A., Gačić, D., Šprem, N., Kamler, J., Baubet, E., Hohmann, U., Monaco, A., Ozoliņš, J., Cellina, S., Podgórski, T., Fonseca, C., Markov, N., Pokorny, B., Rosell, C., Náhlik, A., 2015. Wild boar populations up, numbers of hunters down? A review of trends and implications for Europe: wild boar and hunter trends in Europe. Pest. Manag. Sci. 71, 492–500.

Mattisson, J., Sand, H., Wabakken, P., Gervasi, V., Liberg, O., Linnell, J.D.C., Rauset, G.R., Pedersen, H.C., 2013. Home range size variation in a recovering wolf population: evaluating the effect of environmental, demographic, and social factors. Oecologia 173, 813–825.

Melis, C., Nilsen, E.B., Panzacchi, M., Linnell, J.D.C., Odden, J., 2013. Roe deer face competing risks between predators along a gradient in abundance. Ecosphere 4, 13.

Sand, H., Eklund, A., Zimmermann, B., Wikenros, C., Wabakken, P., 2016. Prey Selection of Scandinavian Wolves: Single Large or Several Small? PLOS ONE 11, e0168062.

Sand, H., Wabakken, P., Zimmermann, B., Johansson, Ö., Pedersen, H.C., Liberg, O., 2008. Summer kill rates and predation pattern in a wolf–moose system: can we rely on winter estimates? Oecologia 156, 53–64.

Ueno, M., Solberg, E.J., Iijima, H., Rolandsen, C.M., Gangsei, L.E., 2014. Performance of hunting statistics as spatiotemporal density indices of moose (*Alces alces*) in Norway. Ecosphere 5, art13.

Wikenros, C., Zimmermann, B., Sand, Håkan, Månsson, Johan, Maartmann, E., Eriksen, A., Wabakken, Petter, 2019. Tildelt, sett og felt elg i forhold til ulveforekomst i Norge og Sverige. Skriftserien nr. 22 - 2019, Høgskolen i Innlandet, Elverum.

Zimmermann, B., Sand, H., Wabakken, P., Liberg, O., Andreassen, H.P., 2015. Predator-dependent functional response in wolves: from food limitation to surplus killing. J. Anim. Ecol. 84, 102–112.
